# Estrogen receptor α (ERα) indirectly induces transcription of human renal organic anion transporter 1 (OAT1)

**DOI:** 10.14814/phy2.14229

**Published:** 2019-11-14

**Authors:** Anna M. Euteneuer, Tamina Seeger‐Nukpezah, Hendrik Nolte, Maja Henjakovic

**Affiliations:** ^1^ Department I of Internal Medicine and Center for Integrated Oncology University of Cologne Cologne Germany; ^2^ Institute of Genetics and Cologne Excellence Cluster on Cellular Stress Responses in Aging‐Associated Diseases (CECAD) University of Cologne Cologne Germany

**Keywords:** Organic anion transporter, OAT1, transcriptional regulation, Estrogen receptor, HNRNPK, CBF

## Abstract

Organic anion transporter 1 (OAT1) is a polyspecific transport protein located in the basolateral membrane of renal proximal tubule cells. OAT1 plays a pivotal role in drug clearance. Adverse drug reactions (ADR) are observed more frequently in women than in men, especially ADR are higher in women for drugs which are known interactors of OAT1. Sex‐dependent expression of Oat1 has been observed in rodents with a tendency to male‐dominant expression. This study aims at elucidating the transcriptional regulation of human OAT1 and tests the effect of estrogen receptor α (ERα). Promoter activation of OAT1 was assessed by luciferase assays carried out by *Opossum* kidney (OK) cells, transiently transfected with promoter constructs of human OAT1 and expression vectors for ERα and exposed to 100 nmol/L 17β‐estradiol. Furthermore, a transcription factor array and proteomic analysis was performed to identify estrogen‐induced transcription factors. Human OAT1 was significantly activated by ligand activated ERα. However, activation occurred without a direct interaction of ERα with the OAT1 promoter. Our data rather show an activation of the transcription factors CCAAT‐box‐binding transcription factor (CBF) and heterogeneous nuclear ribonucleoprotein K (HNRNPK) by ERα, which in turn bind and initiate OAT1 promoter activity. Herewith, we provide novel evidence of estrogen‐dependent, transcriptional regulation of polyspecific drug transporters including the estrogen‐induced transcription factors CBF and HNRNPK.

## Graphical abstract

We provide novel evidence of estrogen receptor‐dependent, transcriptional regulation of organic anion transporter 1. Human OAT1 promoter was indirectly activated by ERα via the transcription factors CBF and HNRNPK.

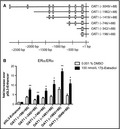

## Introduction

The polyspecific transport protein organic anion transporter 1 (OAT1) contributes to renal clearance by transporting negatively charged endogenous metabolites and exogenous substances, for example, therapeutic drugs or environmental toxins (Burckhardt, 2012). OAT1 transports substrates via a tertiary active transport mechanism at the basolateral side of the proximal tubule cells in the nephron (Burckhardt, 2012). Among other members of solute carrier 22A gene family, OAT1 (SLC22A6) is classified as clinically relevant drug transporter by the FDA (Giacomini et al., 2010). OAT1 has been shown to interact *in vivo* and *in vitro* with various drug classes like nonsteroidal anti‐inflammatory drugs, diuretics, anti‐neoplastics, and antibiotics (VanWert et al., 2010). In clinical observations of patients taking therapeutic drugs, which are OAT1 substrates, higher adverse drug reactions (ADR) in women than in men were identified (Zopf et al., 2009; Rodenburg et al., 2012; Blumenthal et al., 2017; Sharif‐Askari et al., 2017). Taking diclofenac, penicillin, and low ceiling diuretics caused more ADR in women suggesting a sex‐dependent expression of OAT1 in humans (Zopf et al., 2009; Rodenburg et al., 2012; Blumenthal et al., 2017; Sharif‐Askari et al., 2017). In human kidneys, sex‐dependent OAT1 mRNA and protein levels have not been identified so far. Differences in OAT1 mRNA and protein levels have been observed in rodents, being higher in male than in female animals (Cerrutti et al., 2002; Buist and Klaassen, 2004; Wegner et al., 2012).

In contrast to studies focusing on the different mRNA and protein levels of OAT1, our focus is the sex‐dependent transcriptional regulation. Due to the male‐dominant expression of Oat1 in rodents the involvement of androgen receptor(AR)/testosterone complexes was tested (Wang et al., 2012). Previous studies did not state a direct role of ligand‐activated AR in rat Oat1 promoter activation (Wang et al., 2012). However, the transcription factor B‐cell CLL/lymphoma 6 (BCL6) was identified to be male‐dominantly expressed and capable of initiating rat and also human Oat1/OAT1 promoter (Wegner et al., 2012; Wegner et al., 2014). BCL6 induced human OAT1 promoter activation did not occur via BCL6 binding to the promoter region of OAT1 but via hepatocyte nuclear factor (HNF)1α, a known OAT1 regulator (Wegner et al., 2014). BCL6 triggered higher HNF1α protein levels which in turn led to an increased OAT1 promoter activation (Wegner et al., 2014).

The effect of the counterpart to AR, the estrogen receptor (ER)/estrogen transcription factor complex, on OAT1 promoter activation remains to be determined. ER has been shown to be involved in transcriptional regulation of many proteins including also SLC family members, for example, OCTN2 (SLC22A5), a poly‐specific organic cation transporter also expressed in kidneys, which was shown to be regulated by ER in breast cancer cells (Wang et al., 2012). ER belong to the family of nuclear steroid receptors and are ligand (17β‐estradiol)‐activated transcription factors (Scobie et al., 2002). ER play pivotal roles in physiological processes in the human body and are not restricted to women or the reproductive organs (Nilsson and Gustafsson, 2011). There are two different ER, ERα and ERβ, which are encoded by genes located on chromosomes 6q25.1 and 14q23‐24.1, respectively (Marino et al., 2006; Nilsson and Gustafsson, 2011). ERα and ERβ slightly differ in structure and behavior (Marino et al., 2006; Nilsson and Gustafsson, 2011). Ligand‐activated (e.g., 17β‐estradiol bound) and dimerized ER (ERα/ERα, ERα/ERβ, or ERβ/ERβ) translocate into the cell nucleus and unfolds its action by binding to estrogen responsive element (ERE) in a gene promoter (Fan et al., 1996; Heldring et al., 2007). A perfect ERE is a palindromic sequence of 5’ – AGGTCA – 3’ separated by three base spacer, but ER was also shown to bind dissent sequences (Mason et al., 2010; Joshi et al., 2011). Genes like complement factor 3 (C3), leptin, vascular epithelial growth factor (VEGF), breast cancer antigen 1 (BRCA1), and angiotensinogen harbor ERE and are influenced by ER (Fan et al., 1996; Klinge, 2001; O’Neil et al., 2001).

The aim of this study was to investigate the effect of 17β‐estradiol activated ERα on human OAT1 promoter. Initiation of promoter activity and binding of ERα to OAT1 promoter sequences were investigated. We show here ERα‐induced activation of OAT1 transcription. This ERα‐induced activation was surprising as no or a suppressive effect was expected due to male‐dominant expression of Oat1 in rodents (Cerrutti et al., 2002; Buist and Klaassen, 2004; Wegner et al., 2012). However, similar to other transcriptions factors (e. g., BCL6), ERα activation of OAT1 promoter also depend on co‐factors. The transcription factors CCAAT enhancer binding protein zeta (CBF) and heterologous ribonucleoprotein K (HNRNPK) were identified as co‐factors mediating ERα‐dependent transcriptional initiation of OAT1 promoter.

## Materials & Methods

### In silico analysis

Putative ER binding sites were predicted using MatInspector, PROMO, MAPPER, and JASPAR up to three kilobases (kb) upstream of the OAT1 transcriptional starting site based on the genomic sequence NT_167190.1.

### Cell culture, transfection, and luciferase assay

*Opossum* kidney (OK) cells were purchased from LGC Standards (European distributor for “ATCC cultures and bioproducts”; ATCC no.: CRL‐1840). OK cells were cultured in Gibco’s MEM medium supplemented with 10 % FCS, 1 % sodium‐pyruvate, and 1% L‐glutamine at 37°C and 5 % CO_2_ humidified atmosphere. For transient transfection, OK cells were seeded in 24‐well plates at a density of 1 x 10^5^ and grown for 20 h prior transfection. Transfection was performed at a confluency of 70 %, with 25 ng *Renilla* luciferase control vector pRL‐TK (Promega), 500 ng OAT1 promoter fragment in pGL3‐Enhancer vector or empty pGL3‐Enhancer vector (Promega), and 100 ng ERα expression vector (pCMV6‐XL4‐ER, OriGene) using Gibco’s DMEM‐HG medium and Lipofectamine 2000 (Life Technology) according to manufacturers’ instructions (Table 1). After 5 h incubation with the transfection mix, growth medium was changed to 100 nmol/L 17β‐estradiol or 0.001 % DMSO supplemented Gibco’s MEM. After further 43 h incubation the cells were harvested and lysed. *Firefly* and *Renilla* luciferase activities were investigated using Dual‐Luciferase Reporter Assay (Promega) according to the manufacturer’s recommendations. *Firefly* luciferase activity was normalized to *Renilla* and data are presented as fold increase over pGL3‐Enhancer activity.

**Table 1:** Plasmids used in transient transfection of *Opossum* Kidney (OK) cells.

Plasmid	Amount used for transfection
pRL‐TK^a^	25 ng/24 well
pGL3‐Enhancer^a^	500 ng/24 well
pGL3‐Enhancer‐65/+88 bp hOAT1	
pGL3‐Enhancer‐198/+88 bp hOAT1^b^	
pGL3‐Enhancer‐198/+88 bp hOAT1 mut 1	
pGL3‐Enhancer‐198/+88 bp hOAT1 mut 2	
pGL3‐Enhancer‐198/+88 bp hOAT1 mut 3	
pGL3‐Enhancer‐342/+88 bp hOT1^b^	
pGL3‐Enhancer‐746/+88 bp hOAT1^b^	
pGL3‐Enhancer‐1419/+88 bp hOAT1^b^	
pGL3‐Enhancer‐1982/+88 bp hOAT1^b^	
pGL3‐Enhancer‐3049/+88 bp hOAT1^b^	
pCMV6‐XL4‐ERα^c^ (estrogen receptor α expression vector)	100 ng/24 well
pCMV6‐XL5‐ERβ^c^ (estrogen receptor β expression vector)	
pSG5‐hnRNPK^d^ (HNRNPK expression vector)	

a Purchased from Promega

b Wegner *et al.* 2014 ( Wegner et al., 2014);

c Purchased from OriGene

d Kind gift from A. Ostareck‐Lederer & D. Ostareck, University Hospital RWTH Aachen (Ostareck et al., 1997); bp: base pairs.

### Transient suppression of gene expression

Small interfering RNAs (siRNA) were generated to suppress gene expression of proteins of interest against OK transcriptome published by Eshbach et al. (2017) (Table 2). Transfection with 7.5 pmol siRNA in co‐transfections with plasmids (24‐well format) and 50 pmol siRNA in solo transfections (6‐well format) were applied with 2 µL and 5 µL Lipofectamine 2000 reagent per well, respectively. Cells were incubated with transfection mix for 5 h followed by change to normal growth medium. After further 43 h of incubation either luciferase assay, RNA isolation, or nuclear protein extraction was performed to assess gene suppression and the effect of gene suppression on OAT1 promoter activity.

**Table 2:** Used siRNA.

Name (gene target)		Sequence (5‘‐3‘)	Supplier
Non‐target	F	UAGCGACUAAACACAUCAA‐TT	Dharmacon siGENOME non‐targeting siRNA #1
	R	UUGAUGUGUUUAGUCGCUA‐TT	
siCBF #1	F	GACAUUUUUGAGUUUUAUG‐TT	Self‐designed against TRINITY_DN16007_c0_g1_i1
	R	CAUAAAACUCAAAAAUGUC‐TT	
siCBF #2	F	GCAAAGGGCAAAGUUUAAU‐TT	Self‐designed against TRINITY_DN16007_c0_g1_i1
	R	AUUAAACUUUGCCCUUUGC‐TT	
siHNRNPK #1	F	GGCCGUGGCUCAUAUGGUG‐TT	Self‐designed against TRINITY_DN32136_c0_g1_i1
	R	CACCAUAUGAGCCACGGCC‐TT	
siHNRNPK #2	F	CCCUAUGAUCCUAAUUUCU‐TT	Self‐designed against TRINITY_DN32136_c0_g1_i1
	R	AGAAAUUAGGAUCAUAGGG‐TT	

F, forward; R, reverse; si, small interfering.

### RNA isolation, reverse transcription, and real‐time PCR

Total RNA isolation from cells was performed using the RNeasy^®^ Mini Kit (Qiagen). Transiently transfected OK cells were washed twice with ice‐cold PBS prior addition of RLT lysis buffer (100 µL for 24‐well, 200 µL for 6‐well) for direct lysis. Lysates were collected in a 1.5 mL micro‐tube, vortexed, and further homogenized by passing the lysate at least five times through a blunt 29‐gauge needle. All subsequent steps were carried out according to the manufacturer’s instructions. RNA quantity and quality were measured on a Nanodrop ND‐1000 spectrometer (Thermo Fisher). Subsequently, 2 µg RNA were reverse transcribed using Superscript^®^ II reverse transcriptase (Invitrogen™) according to manufacturer’s recommendations. RNA, deoxynucleotides (dNTP), oligonucleotides (Oligo dT12 5’‐TTTTTTTTTTTT‐3’), and 0.5 µL Superscript^®^ II reverse transcriptase per sample were mixed and placed in a C1000 Touch Thermal Cycler (Biorad).

Endogenous OK cell gene expression was measured with the GoTaq^®^ qPCR Master Mix (Promega) according to the manufacturer’s instructions. Subsequently, PCR reaction was analyzed on a 7500 fast real‐time PCR system with initial heating phase of 2 min at 95°C, 30 cycles of 95°C for 15 sec, and annealing at 55°C for 1 min. Primers were generated using the published transcriptome sequences of OK cells (Eshbach et al., 2017) (Table 3).

**Table 3:** Primer for double‐stranded DNA‐binding dye real‐time PCR.

Gene		Sequence (5'‐3')	Lengh	Trinity ID
GAPDH	F	GCATCCTGGGCTACACAGAG	107 bp	TRINITY_DN35766_c0_g1_i4
	R	AGAAATGGTCGTTGAGGGCAA		
PPIA	F	CATCTGTACTGCTAAGACTGATTG	126 bp	TRINITY_DN105025_c0_g1_il
	R	CTTCTTGCTGGTCTTGCCATCCC		TRINITY_DN68411_c0_g1_i1
SLC22A6 (OAT1)	F	CGCCTTCTTTCTGCCTGAGA	174 bp	TRINITY_DN29038_c0_g1_i1
	R	TGCCCAACTTCCTGTCATCC		
HNRNPK^a^	F	AATGCCAGTGTTTCAGTCCC	119 bp	TRINITY_DN32136_c0_g1_i1
	R	AGGCCCTCTTCCAAGGTAGG		
CBF	F	ATGCTGGCTACCTTGGATGA	90 bp	TRINITY_DN16007_c0_g1_i1
	R	TGCTTCTAGTTCACCCTGCT		

F, forward; R, reverse.

a Primer sequence adapted from Mikula et al. (2013)*.*

The housekeeping genes GAPDH and PPIA were measured in addition to genes of interest to control mRNA integrity and for quantification. The expression levels of gene of interest were then calculated by the 2^‐∆∆Ct^ method which represents the fold change of the protein of interest relative to its control (Livak and Schmittgen, 2001).

ΔCt=Ct gene of interest-Ct housekeeping gene

ΔΔCt=ΔCt 17β-estradiol-ΔCt DMSO or

ΔΔCt=ΔCt siNT-ΔCt siRNA gene of interest

$$2^{-\Delta \Delta \text{Ct}}=\text{ratio}$$

### Nuclear extraction

Nuclear extracts of transiently transfected OK cells were prepared using either nuclear extraction kit (AY2002, Affymetrix) or NE‐PER™ isolation kit (Thermo‐Fisher) according to the manufacturer’s instructions. Protein content was determined using Bio‐Rad DC Protein Assay kit (PN 500‐0112) (Biorad) or Pierce^TM^ BCA Protein Assay Kit (Thermo‐Fisher), respectively. Prepared nuclear extracts were used for proteomic analysis, transcription factor array, western blot, and electromobility shift assay (EMSA).

### Western blot

Proteins were separated by sodium dodecyl sulfate polyacrylamide gel electrophoresis (SDS‐PAGE). Nuclear and cytoplasmic extracts of OK cells which were transiently transfected with gene expression vectors or siRNA for gene suppression were prepared for western blot analysis as follows: 20 ‐ 40 µg protein was mixed with 6 x Laemmli buffer and H_2_O followed by vortexing and an incubation step of 5 min at 95°C. Samples were loaded on a 10 % polyacrylamide gel with stacking gel and electrophoresis was performed for 20 min at 100 Volt (V) followed by 60 ‐120 min at 120 V in a Mini‐PROTEAN® Tetra Cell Systems (Biorad). Precision Plus Protein™ WesternC™ Blotting Standards was used to detect protein expression according to molecular weight. Proteins were then transferred to a PVDF membrane (GE Healthcare Europe GmbH) with the standard program (30 min at 25 V) of a Trans‐Blot^®^ Turbo™ Transfer System (Bio‐Rad). After blotting, the membranes were blocked using Tris‐buffered saline (TBS) containing 0.1% Tween‐20 and 5 % non‐fat dry milk and membranes were further incubated with primary antibody overnight at 4°C (HNRNPK: sc‐28380, Santa Cruz; UAP56, kind gift from Niels Gehring, Institute for Genetics, University of Cologne (Gromadzka et al., 2016)). After extensive washing with TBST, membranes were incubated with the corresponding secondary HRP‐coupled antibody and protein bands were visualized by enhanced chemiluminescence (ECL) using Pierce^®^ ECL Western Blotting Substrate and SuperSignal™ West Femto Maximum Sensitivity Substrate (ratio 4:1) on an ECL ChemoCam Imager workstation (INTAS Science Imaging Instruments GmbH). Semi‐quantification of protein expression was performed using ImageJ software.

### Electromobility shift assay (EMSA)

Electromobility shift assay (EMSA) was performed with LightShift™ Chemiluminescent EMSA Kit (Thermo Fisher). Nuclear extracts were applied in concentrations of 1 and 3 µg for exogenous and endogenous protein expression, respectively. A mixture of isolated nuclear extract, 20fmol biotin‐labeled oligonucleotides (Table 4), 50 ng/µL poly‐dIC, 2.5 % glycerol, 5 mmol/L MgCl_2,_ 0.05 % NP‐40, and 2 µL of 10 x binding buffer was incubated in a total volume of 20 µL for 20 min at room temperature prior loading on a 6 % polyacrylamide gel. Electrophoresis at 100 V was performed until the bromphenol blue dye had migrated approximately two‐thirds down the gel followed by blotting on a nylon membrane (Roche) for 30 min at 380 V and crosslinking by UV‐light for 2 min. Finally, membrane was exposed to an ECL ChemoCam Imager workstation (INTAS Science Imaging Instruments GmbH) and the biotin‐labeled DNA‐protein complexes were visualized. Image editing was performed with Image J. For supershift assay 0.1 µg ERα (sc‐8002x, Santa Cruz) and 1 µg HNRNPK (sc‐28380, Santa Cruz) antibody were added to the samples.

**Table 4:** Used Oligonucleotides in EMSA.

Name/Position	Database	Protein (Gene)		Sequence (5' ‐ 3')
B‐ERE consensus	Mason et al. (2010)	ERα (ESR1)	F	GGATCTAGGTCACTGTGACCCCGGATC
			R	GATCCGGGGTCACAGTGACCTAGATCC
B‐OAT1 ‐198/‐165	NT_167190.1	OAT1 (SLC22A6)	F	GGTCCAATAGATCCCACTCTGGCCCCCCCTGCCC
			R	GGGCAGGGGGGGCCAGAGTGGGATCTATTGGACC
B‐OAT1 ‐198/‐165 mut	NT_167190.1	OAT1 (SLC22A6)	F	GGTCCAATAGATCCCACTCTGGC**AA**C**AA**CTGCCC
			R	GGGCAG**TT**G**TT**GCCAGAGTGGGATCTATTGGACC
B‐OAT1 ‐175/‐150	NT_167190.1	OAT1 (SLC22A6)	F	CCCCCCTGCCCCCAGATGCCCCCCT
			R	AGGGGGGCATCTGGGGGCAGGGGGG
B‐OAT1 ‐164/‐131	NT_167190.1	OAT1 (SLC22A6)	F	CCAGATGCCCCCCTAATACACCCCTCTCCCTGCT
			R	AGCAGGGAGAGGGGTGTATTAGGGGGGCATCTGG
B‐OAT1 ‐142/‐117	NT_167190.1	OAT1 (SLC22A6)	F	CCTCTCCCTGCTCCTATTCAGTCCA
			R	TGGACTGAATAGGAGCAGGGAGAGG
B‐OAT1 ‐130/‐97	NT_167190.1	OAT1 (SLC22A6)	F	CCTATTCAGTCCACCCTCTCCTGCCCTTTATAAC
			R	GTTATAAAGGGCAGGAGAGGGTGGACTGAATAGG
B‐OAT1 ‐108/‐83	NT_167190.1	OAT1 (SLC22A6)	F	GCCCTTTATAACCACTTGGAGAAAT
			R	ATTTCTCCAAGTGGTTATAAAGGGC
B‐OAT1 ‐96/‐63	NT_167190.1	OAT1 (SLC22A6)	F	CACTTGGAGAAATTCCACTGACACAAGGAATCC
			R	GGATTCCTTGTGTCAGTGGAATTTCTCCAAGTG
B‐OAT1 ‐78/‐53	NT_167190.1	OAT1 (SLC22A6)	F	TGACACAAGGAATCCTTGGAGGGTT
			R	AACCCTCCAAGGATTCCTTGTGTCA
B‐OAT1 ‐66/‐34^a^	NT_167190.1	OAT1 (SLC22A6)	F	TCCTTGGAGGGTTAATCCTTCTGATACCAAGTC
			R	GACTTGGTATCAGAAGGATTAACCCTCCAAGGA

Bold and underscored nucleotides indicate mutations. F, forward; R, reverse.

a Wegner et al. (2014).

### Transcription factor array

Transcription factors activated upon 17β‐estradiol incubation were identified by Transcription Factor Activation Profiling Plate Array I (Signosis). This array is a multiplex assay testing 48 different transcription factors at once based on hybridization. Nuclear extract of OK cells (10 µg), transiently transfected with ERα (pCMV6‐XL4‐ERα) expression vector, were used. The two conditions of 100 nmol/L 17β‐estradiol and 0.001 % DMSO were compared. The experimental procedure was done according to the manufacturer’s instructions and the plates were read on a Paradigm^®^ detection platform (Beckman Coulter).

### Sample preparation and protein digest for proteomic analysis

Nuclear extract from ERα transfected and 17β‐estradiol or DMSO incubated OK cells was prepared and 30 µg of nuclear extract per sample were diluted in a buffer containing, 4 % SDS in 100 mmol/L Tris‐HCl (pH 7.6). Subsequently, acetone precipitation of proteins was performed by adding 4 volumes of acetone to the samples followed by an overnight incubation at ‐20°C. On the next day, samples were centrifuged for 15 min at full speed in a benchtop centrifuge. Supernatant was removed, and the pellet was washed twice with 1 mL ice‐cold acetone and 10 min centrifugation step at full speed. The pellet was then air dried and reconstituted in 20 µL 8 M urea‐lysis‐buffer for in solution digest. Dithiothreitol (DTT) was added to the samples to reach a final concentration of 5 mmol/L DTT and the samples were incubated for 1 h at 37°C. Subsequently, Chloracetamide was added to the samples to a final concentration of 40 mmol/L followed by vortexing and a 30‐min incubation in the dark. To digest the proteins, endoproteinase Lys‐C (Wako) was added to the samples with an enzyme:substrate ratio of 1:75 and incubated at 37°C for 4 h. After Lys‐C digest, the samples were further digested with trypsin. Therefore, the samples were diluted in 50 mmol/L TEAB to achieve a urea concentration of 2 M before trypsin was added with an enzyme:substrate ratio of 1:75 and overnight incubation at 37°C. The digest was stopped by acidification by adding formic acid to a final concentration of 1%. Purification of sample peptides was performed by stop and go extraction (STAGE) Tips (Rappsilber et al., 2003).

### Ultra‐high‐pressure Liquid chromatography and mass spectrometry

Samples were measured on a nanoLC coupled via nano electrospray ionization to the QExactive HF‐x benchtop instrument by the Proteomics Facility of the CECAD Institute (Cologne). In brief, samples were analyzed after in‐solution digestion using a 90 min gradient with linearly increasing concentrations of solvent B for 65 min, followed washing at 95% B and 5 min re‐equilibration to 5% solvent B on a 50 cm column (i.d. 75 μm), packed in house with 1.9 μm C18 beads (Dr. Maisch, Germany). For label free quantitation, spectra were acquired at a resolution of 70 000 at 200 m/z. An automated gain control (AGC) target of 3 x 10^6^ and a maximum injection time of 20 msec were used. Tandem mass spectrometry (MS/MS) spectra were acquired in a top 22 data‐dependent mode using a resolution of 15 000 at 200 m/z after accumulation of 5 × 10^5^ AGC targets within an injection time of 20 ms. Ions were isolated at a 1.3 Th isolation window and fragmented in the HCD cell at 27 normalized energy. Dynamic exclusion was set to 25 sec.

### Raw data processing and data analysis

Raw files were processed with MaxQuant (1.5.3.8). Electrospray (ESI)‐MS/ MS spectra were correlated for protein assignment to the 6 frame translated transcriptomics database by Eshbach et al. (2017) using the implemented Andromeda Search engine. Proteins were further verified by blast against human and rat proteome. A list of common contaminants was included for filtering. Mass tolerances for MS/MS spectra in first search were set to 20 ppm and for main search to 4.5 ppm. Acetylation at the N‐term (Protein) and oxidation of methionine was set as a variable modification, while carbamidomethylation at cysteine residues was considered as a fixed modification. Peptidespectra‐match false discovery rate (FDR) and protein FDR was set to 0.01, using the decoy algorithm in revert mode (MaxQuant). Proteins were quantified by the implemented label‐free quantification algorithm (MaxLFQ). Intensities were log2 transformed and significant differently expressed proteins were detected by applying a two‐sided t‐test. Correction for multiple comparison was done by estimating the FDR by a permutation approach to 5%. Data were visualized using Instant Clue Software (Nolte et al., 2018).

### Site‐directed mutagenesis of predicted ER‐binding sites

QuickChange^®^ II site‐directed mutagenesis kit (Agilent Technologies) was used to insert point mutations into predicted binding sites to subsequently prevent transcription factor binding. The online tool MAPPER (http://bio.chip.org/mapper) was used to verify destruction of the predicted binding site. Constructs harboring more than one mutation were generated by consecutive rounds of mutagenesis. Primers for mutation insertion (Table 5) were designed with the online tool QuickChange^®^ Primer Design (https://www.genomics.agilent.com/primerDesignProgram.jsp) against predicted ERα binding sited in OAT1 promoter. Mutagenesis was performed according to manufacturer’s recommendation and the product of the mutagenesis‐PCR was transformed into XL1‐Blue supercompetent cells. After overnight incubation of plated XL1‐Blue supercompetent cells, three clones were picked and a miniprep with QIAprep^®^ Spin Miniprep Kit (Qiagen) was performed followed by sequencing to ensure successful mutation. Clones harboring the new mutations were amplified as described in Plasmid amplification. Sequencing was performed with BigDye^®^ terminator v1.1 cycle sequencing kit (Thermo Fisher) to verify plasmid mutation. For sequencing a forward and reverse primer starting in the pGL3‐Enhancer sequence neighboring the insert was used: forward 5’‐ CTGTGTGTTGGTTTTTTGTGTG −3’, reverse 5’‐ TCTCCAGCGGTTCCATCTTC −3’. The PCR reaction was set up in 10 µL with 0.5 µL BigDye^®^, 2 µL sequencing buffer, 350–400 ng plasmid and 10 pmol primer and performed with 5 min initial denaturation at 96°C followed by denaturation at 96°C for 10 sec, annealing at 55°C for 15 sec, and elongation at 60°C for 4 min for 25 cycles before handing in the samples at the Cologne Center for Genomics (CCG) for analysis. Cleaning was performed by the facility. Data analysis was performed with Chromas lite software.

**Table 5:** Site‐directed mutagenesis primer for pGL3‐Enhancer‐198/+88 bp hOAT1.

Name		Sequence (5' ‐ 3')
Mut 1	F	TTCAGTCCACCCTCTCCTGC**T**CT**C**T**G**TAACCACTTGGAGAAATTCC
	R	GGAATTTCTCCAAGTGGTTA**C**A**G**AG**A**GCAGGAGAGGGTGGACTGAA
Mut 2	F	TAAAGGGCAGGAGAGGGCT**GA**CTGAATAGGAGCAGG
	R	CCTGCTCCTATTCAG**TC**AGCCCTCTCCTGCCCTTTA
Mut 3	F	TA**C**A**G**AG**A**GCAGGAGAGGG**GA**GA**AA**GAATAGGAGCAGGGAGAGG
	R	CCTCTCCCTGCTCCTATTC**TT**TC**TC**CCCTCTCCTGC**T**CT**C**T**G**TA

Bold and underscored nucleotides indicate mutations. F, forward; R, reverse;

### Statistics

For statistical analysis the unpaired, two‐sided *student’s t‐test* was used which was calculated by GraphPad Prism or Microsoft Excel. Data are presented as means ± standard error of mean (SEM) with ***: *P* ≤ 0.001; **: *P* ≤ 0.01; * *P* ≤ 0.05; ns: not significant.

## Results

### *Activation of OAT1 promoter by ER*α

**Figure 1:**
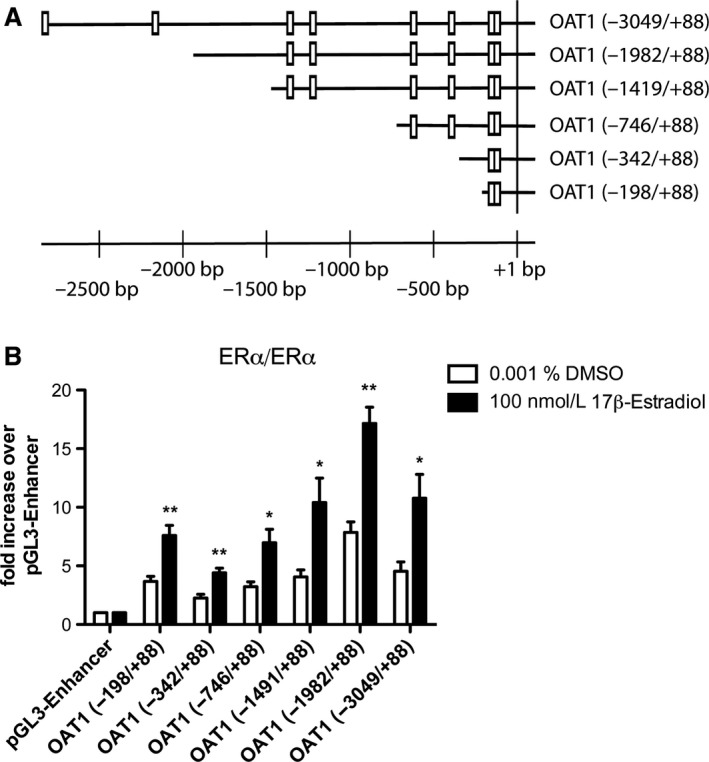
Activation of organic anion transporter 1 (OAT1) by ERα. **(A)** Localization of predicted ER binding sites in different lengths of human OAT1 promoter indicated by boxes. bp: base pairs; +1: transcriptional starting site. **(B)** OK cells were transiently transfected with indicated human OAT1 promoter construct or pGL3‐Enhancer and ER expression plasmid followed by incubation with 100 nmol/L 17β‐estradiol or DMSO control for 43 h prior luciferase assay. Data are normalized to *Renilla* luciferase expression, reported as fold increase over pGL3‐Enhancer and presented as mean ± S.E.M.; *n* = 4; *: *P* < 0.05; **: *P* < 0.01 significantly different to DMSO control using the unpaired two‐tailed *t‐test*.

Several cloned 5’‐truncated promoter fragments of OAT1 which differ in length and number of putative ER‐binding sites were used (Fig. 1A). All tested OAT1 promoter fragments were significantly activated by the ERα/ERα homodimer, showing a twofold increase in promoter activity (Fig. 1B). In addition, no OAT1 promoter activation was found in the presence of ligand‐activated ERα/ERβ heterodimer and ERβ/ERβ homodimer (Figure S1, https://doi.org/10.6084/m9.figshare.9118331).

### ERα does not bind to human OAT1 promoter

**Figure 2:**
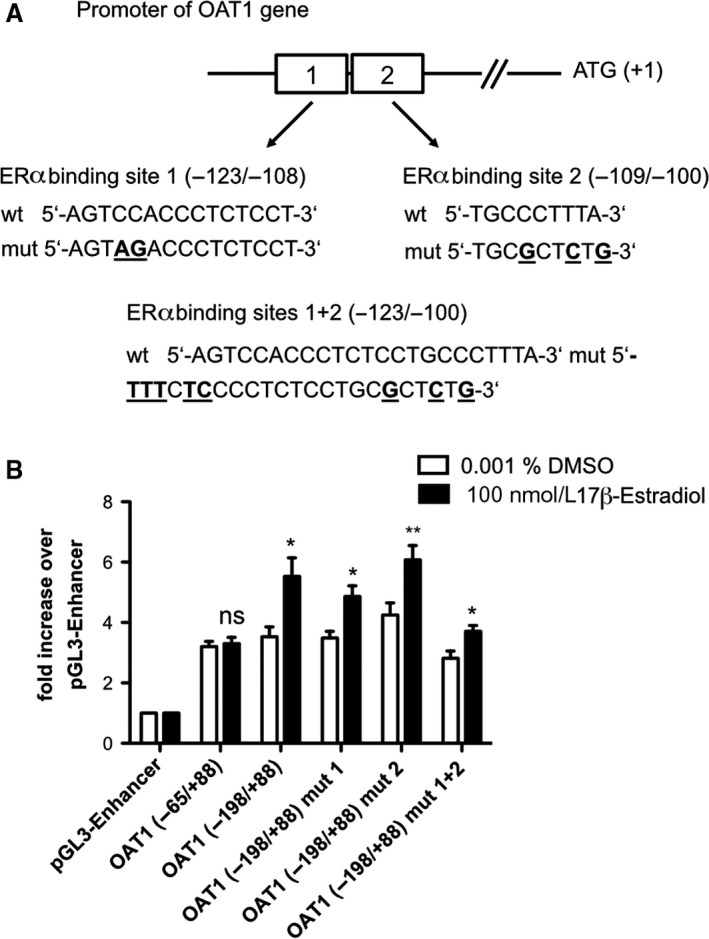
ERα does not bind to human OAT1 promoter. **(A)** Localization of predicted ER binding sites in OAT1 promoter fragment indicated by boxes. **(B)** OK cells were transiently transfected with wt or mutated −198/+88 OAT1 promoter construct, −65/+88 OAT1 promoter construct or pGL3‐Enhancer and ERα expression plasmid followed by incubation with 100 nmol/L 17β‐estradiol or DMSO control for 43 h prior luciferase assay. Data are normalized to *Renilla* luciferase (pRL‐TK) expression, reported as fold increase over pGL3‐Enhancer and presented as mean ± S.E.M.; *n* ≥ 4; *: *P* < 0.05, **: *P* < 0.01; ns: not significant using the unpaired two‐tailed *t‐test*

OAT1 (−342/+88) promoter construct showed no additional enhancement of promoter activation by ERα which suggests that ERα binds within −198 to +88 bp. Two ER binding sites were predicted within the OAT1 promoter fragment −198/+88 bp, lying −123/−108 and −109/−100 bp before the transcriptional starting site (TSS) (Fig. 2A). Subsequent testing of an OAT1 promoter fragment (−65/+88 bp) without predicted estrogen responsive elements resulted in no activation of OAT1 promoter fragment by ligand induced ERα (Fig. 2B). The predicted ER binding sites −123/−108 and −109/−100 in the −198/+88 OAT1 promoter construct were modified by introducing point mutations (Fig. 2A). Surprisingly, ligand‐induced ERα activation of OAT1 promoter was not affected by mutations in the predicted binding sites (Fig. 2B). Additionally, binding of ERα/ERα homodimer to OAT1 promoter was investigated using electromobility shift assay (EMSA). Eight biotin (B)‐labeled oligonucleotides containing OAT1 promoter fragments spanning −198 to −53 bp before TSS were used, but no ERα protein–DNA complexes were observed (Figure S2, https://doi.org/10.6084/m9.figshare.7805990).

### Ligand‐activated ERα induces the activity of transcription factor CBF in OK cells

No binding of ERα to OAT1 promoter suggests that ERα induces other proteins, especially transcription factors, which in turn would activate the OAT1 promoter. Therefore, a transcription factor activation profiling array was performed to identify transcription factors that could be involved in ERα‐induced OAT1 promoter activation.

In this array, 48 different transcription factors were measured within a nuclear extract of OK cells transfected with ERα expression plasmid and after exposure to 17β‐estradiol or DMSO as control for 43 h.

In parallel, ERα‐dependent activation of the C3 promoter was determined in luciferase assay, serving as positive control to ensure successful ERα stimulation in OK cells (Figure S3, 10.6084/m9.figshare.9118340).

**Figure 3:**
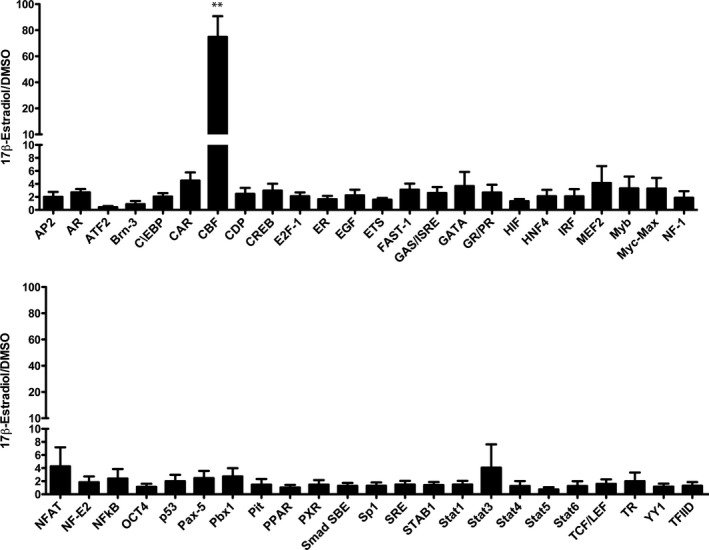
**Ligand‐activated ERα induces transcription factor CBF.** Nuclear extracts of OK cells transiently transfected with ERα expression plasmid and either incubated with 17β‐estradiol or DMSO control for 43 h prior nuclear extract preparation were tested with a transcription factor activation profiling plate array (Signosis). Data are presented as ratio of 17β‐estradiol/DMSO with ± S.E.M.; *n* = 4.

**Figure 4:**
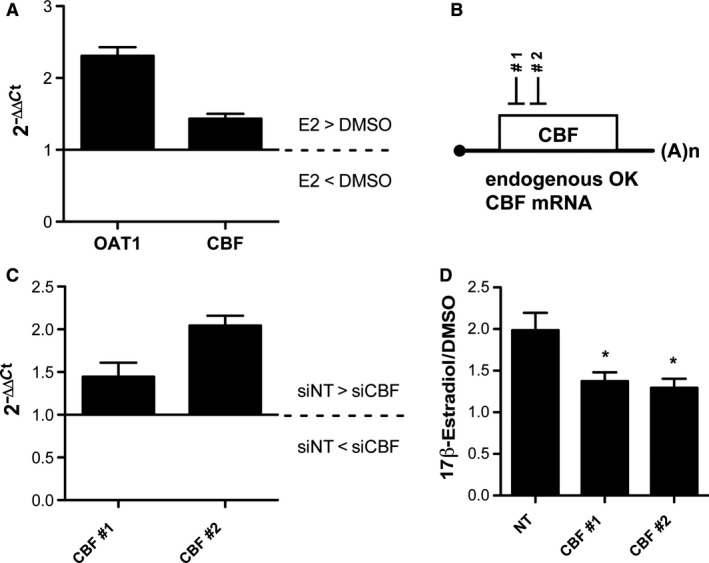
ERα indirectly activates OAT1 by ERα‐induced CBF activity.** (A)** RNA was isolated from OK cells transiently transfected with ERα expression vector. Endogenous OK cell OAT1 and CBF mRNA level were compared between 17β‐estradiol (E2) and DMSO incubated cells. Data are presented as 2^−ΔΔCt^. *n* = 4. **(B)** siRNAs were designed against endogenous CBF mRNA in OK cells. **(C)** In siRNA‐transfected OK cells, CBF expression was investigated using real‐time PCR. Data are presented as 2^−ΔΔCt^. **(D)** Promoter activity of −198/+88 OAT1 promoter construct was determined after OK cell transfection with ERα expression vector and either with non‐target (NT) or siRNA against CBF (CBF #1, CBF #2) and incubation with 100 nmol/L 17β‐estradiol or DMSO control for 43 h. Data are normalized to *Renilla* luciferase expression and are presented as ratio of 17β‐estradiol to DMSO with mean ± S.E.M.; n_CBF #1_ = 16; n_CBF #2_ = 12; *: *P* < 0.05 significantly different to NT.

The transcription factor CCAAT enhancer binding protein zeta (CBF or C/EBPζ) was the only out of 48 factors which showed significantly (p < 0.01) upregulated activity in 17β‐estradiol incubated cells (Fig. 3). The involvement of CBF in ERα‐induced OAT1 promoter activation was ascertained by additional experiments. Endogenous CBF mRNA of OK cells was significantly (*P* < 0.05) induced upon 17β‐estradiol incubation. Additionally, endogenous OAT1 mRNA level was also increased (*P* < 0.01) by ERα transfection and 17β‐estradiol (Fig. 4A). Small interfering RNAs (siRNA) were generated against endogenous CBF mRNA to suppress gene expression (Fig. 4B). Both generated siRNAs – CBF #1 and CBF #2 – significantly reduced CBF mRNA expression in OK cells (Fig. 4C). In addition, significantly reduced activation of OAT1 promoter fragment (−198/+88) was observed in luciferase assays when CBF #1 and CBF #2 were applied (Fig. 4D).

### Ligand‐activated ERα induces transcription factor HNRNPK activity

In addition to the transcription factor array, mass spectrometry analysis of ERα transfected and 17β‐estradiol or DMSO incubated OK cells was performed to identify proteins responsible for ERα‐induced OAT1 promoter activation. To ensure successful stimulation of ERα, a luciferase assay with the ERα‐induced C3 gene promoter (positive control) was performed in parallel which clearly showed a significant increase on C3 promoter activation in 17β‐estradiol incubated cells (Figure S3).

**Figure 5:**
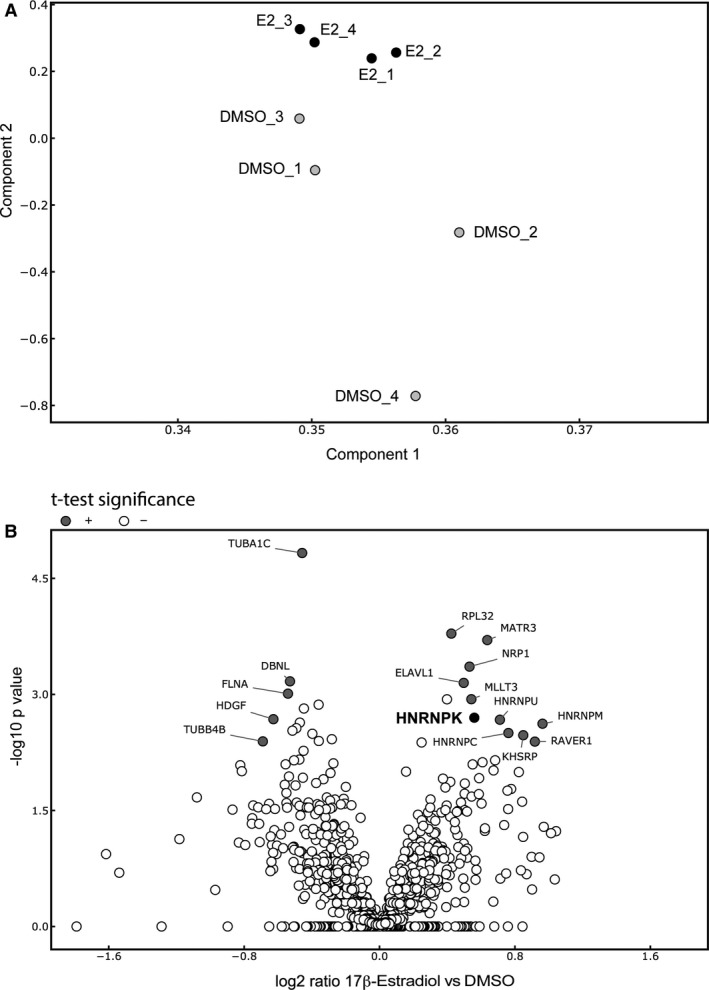
ERα induces transcription factor heterologous ribonucleoprotein K (HNRNPK). OK cells were transfected with ERα expression plasmid and either cultured in 17β‐estradiol or DMSO control for 43 h prior nuclear extract preparation and mass spectrometry analysis on a QExactive Plus mass spectrometer. Data are visualized with Instant Clue software. **(A)** Principle component analysis (PCA) of mass spectroscopy analysis. **(B)** Volcano plot plotting the log2 fold difference from 17β‐estradiol versus DMSO control incubated samples against the ‐log_10_ *P*‐value. +: significantly up or downregulated proteins (FDR 5 %).

To obtain a systematic view on the data, a principal component analysis (PCA) was utilized and revealed a clear segregation of samples stimulated by 17β‐estradiol and DMSO control cells (Fig. 5A). This indicates that 17β‐estradiol results in a unique protein expression signature. Differentially expressed proteins upon 17β‐estradiol incubation in OK cells were identified by two‐sided *t‐test* with correction for multiple testing by a randomization‐based false discovery rate (FDR) calculation (cutoff 5%). Results were plotted in a volcano plot identifying 11 significantly up‐ and 5 downregulated proteins upon 17β‐estradiol incubation (Fig. 5B). Among the significantly upregulated proteins, the heterologous ribonucleoprotein K (HNRNPK) (p‐value: 0.002) caught our attention due to its ability to act as transcription factor (Michelotti et al., 1996).

**Figure 6:**
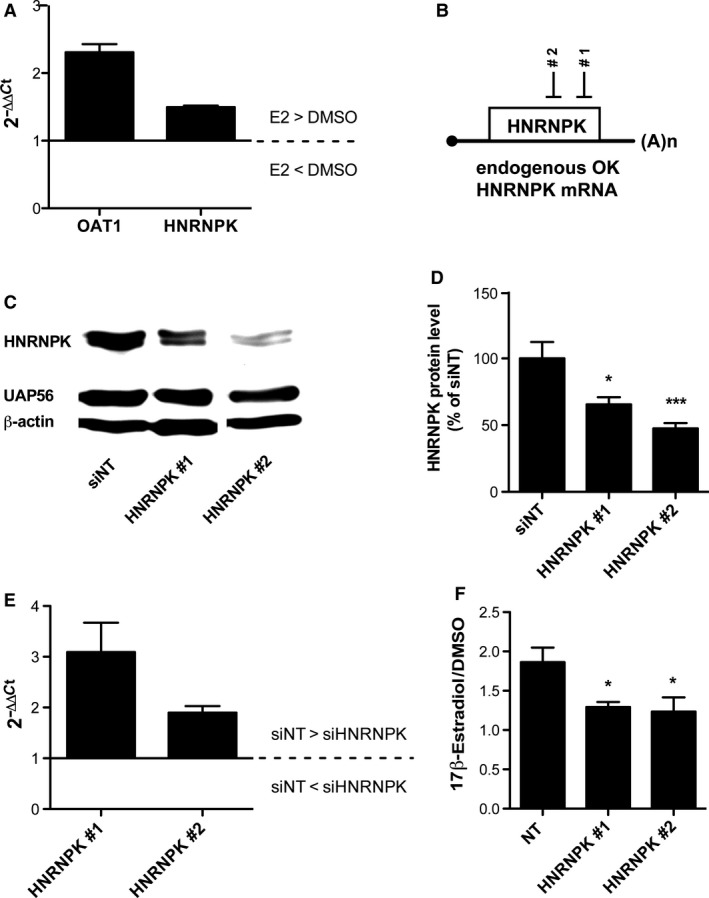
ERα indirectly activates OAT1 by ERα‐induced HNRNPK activity.** (A)** RNA was isolated from OK cells transiently transfected with ERα expression vector. Endogenous OK cell OAT1 and HNRNPK mRNA level were compared between 17β‐estradiol (E2) and DMSO incubated cells. Data are presented as 2^−ΔΔCt^. *n* = 4. **(B)** siRNA were designed against endogenous OK cell mRNA. **(C)** After transfection with siRNAs, HNRNPK protein level was determined by western blot. **(D)** HNRNPK protein expression was semi‐quantified with Image J. HNRNPK expression was normalized to expression of nuclear protein UAP56. Normalized siNT expression was set to 100% and data are presented as mean S.E.M; *:*P* < 0.05; **P < 0.01 significantly different from siNT with unpaired two‐sided *t‐test.* *n* = 10. **(E)** RNA was isolated from transiently siRNA transfected OK cells and HNRNPK mRNA level was determined. Data are presented as 2^−ΔΔCt^. *n* = 8**. (F)** OK cells were transfected with −198/+88 OAT1 promoter construct, ERα expression vector and either with non‐target (NT) or gene specific siRNA. Subsequently, cells were incubated with 100 nmol/L 17β‐estradiol or DMSO control for 43 h prior luciferase assay. Data are normalized to *Renilla* luciferase (pRL‐TK) expression and are presented as ratio of 17β‐estradiol to DMSO with mean ± S.E.M.; n_HNRNPK #1_ = 16; n_HNRNPK #2_ = 12; *: *P* < 0.05.

The involvement of HNRNPK in ERα induced OAT1 promoter activation was ascertained by additional experiments. HNRNPK and OAT1 mRNA levels were significantly (*P* < 0.01) induced in OK cells upon 17β‐estradiol incubation (Fig. 6A). Both generated siRNAs against endogenous HNRNPK significantly reduced HNRNPK mRNA and protein level (Fig. 6C–E). As expected, 17β‐estradiol‐activated ERα‐induced OAT1 promoter activation was reduced significantly in luciferase assays when HNRNPK expression was suppressed by applying HNRNPK #1 and HNRNPK #2 (Fig. 6F).

### HNRNPK binds OAT1 promoter

**Figure 7:**
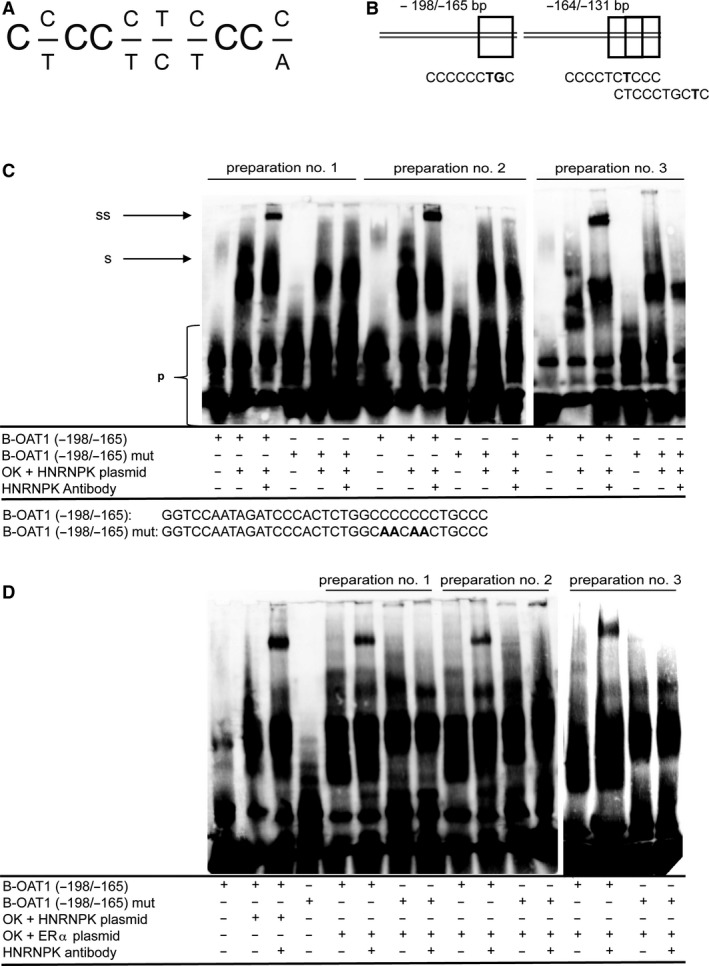
Exogenous and endogenous HNRNPK binding in OAT1 promoter. **(A)** HNRNPK binding motif (Mikula et al., 2010). **(B)** Biotin (B)‐labeled oligonucleotides −198/−165 and −164/−131 of human OAT1 promoter. Boxes emphasize predicted HNRNPK binding sites. Bold nucleotides indicate dissimilarity to HNRNPK binding motif. **(C)** Nuclear extracts (1 µg) of OK cells transfected with HNRNPK expression vector were mixed with biotin‐labeled (−198/−165) OAT1 promoter fragment or a mutant (−198/−165) OAT1 probe in the presence or absence of HNRNPK antibody. Three independent preparations are shown. p: unbound probe; s: shift; ss: super‐shift. **(D)** Nuclear extracts (1 µg) of OK cells transfected with HNRNPK expression vector were mixed with biotin‐labeled (−198/−165) OAT1 promoter fragment in the presence or absence of HNRNPK antibody as positive control. Nuclear extracts (3 µg) of OK cells transfected with ERα expression vector and incubated with 17β‐estradiol for 43 h prior nuclear extract preparation were incubated with biotin‐labeled OAT1 (−198/−165) probe and a mutant version in EMSA. Three independent preparations are shown. p: unbound probe; s: shift; ss: super‐shift.

Whether HNRNPK is binding directly to the proximal OAT1 promoter was further investigated. Mikula *et al.* published a binding site motif for HNRNPK (Fig. 7A) (Mikula et al., 2010). OAT1 promoter was scanned manually to predict sites for HNRNPK binding. Three binding sites (highlighted with boxes) were identified by screening of proximal OAT1 promoter: −176/−166, −144/−132, and −139/−129 (Fig. 7B). Therefore, the biotin‐labeled OAT1 promoter fragments −198/−165 and −164/−131 were used in EMSA with a nuclear extract, which was prepared from HNRNPK expression plasmid transfected OK cells (Ostareck et al., 1997). Application of the biotin‐labeled OAT1 promoter fragment −198/‐165 resulted in HNRNPK‐specific shifts and super‐shifts in three independent experiments (Fig. 7C). Neither a shift nor a super‐shift was observed when a mutated biotin‐labeled OAT1 probe was applied (Fig. 7C). Despite two predicted HNRNPK binding sites, EMSA with the OAT1 promoter sequences −164/−131 showed no shifted or super‐shifted bands (Figure S4, 10.6084/m9.figshare.7806035). In addition to exogenous HNRNPK binding to the biotin‐labeled OAT1 promoter fragment (−198/−165), endogenous ERα‐induced HNRNPK binding to OAT1 promoter to the wild type but not the mutant probe was also shown (Fig. 7D). Therefore, nuclear extracts from OK cells transfected with ERα and incubated with 17β‐estradiol were used (Fig. 7D). In addition, nuclear extracts from cells transiently transfected with siRNA HNRNPK #1 and HNRNPK #2 were used in EMSA (Figure S5, https://doi.org/10.6084/m9.figshare.7806035). Clear shifted and super‐shifted bands were observed for the biotin‐labeled probe B‐OAT1 (−198/−165) when incubated with nuclear extract from non‐target siRNA (siNT) treated cells whereas a reduction in HNRNPK‐specific shifted and super‐shifted band intensity was observed for nuclear extracts which were prepared from OK cells transfected with siRNA HNRNPK #1 and #2 (Figure S5).

## Discussion

Human OAT1 are crucial for secretion of endo‐ and exogenous organic anions and deficiencies or inhibition interferes with this task (Eraly et al., 2006; Wang and Sweet, 2013). About 306 OAT1 substrates, but only a handful of transcription factors regulating OAT1 are known today (Kikuchi et al., 2007; Saji et al., 2008; Jin et al., 2012; Wegner et al., 2014). In this study, the involvement of ERα in OAT1 transcriptional regulation was tested for the first time. ERα is present in human and mouse proximal tubule cells (Grimont et al., 2009; Irsik et al., 2013; El‐Deek et al., 2018). A study of Jelinsky and colleagues demonstrated that mice kidney is at third place of organs responding to estradiol stimulus and ERα was shown to regulate gene expression of various genes in mouse kidney (Jelinsky et al., 2003).

Our data show that ligand activated ERα did significantly activate all tested human OAT1 promoter fragments. The activation of OAT1 promoter by 17β‐estradiol induced ERα was surprising due to the generally accepted male‐dominant expression of Oat1 in rodents (Ljubojević et al., 2004; Wegner et al., 2012; Breljak et al., 2013). In most cases, ligand‐activated and dimerized ERα binds directly to its DNA binding motif consisting of the palindromic consensus sequence “AGGTCAnnnTGACCG”. Surprisingly, insertion of mutations into the predicted binding sites −123/−108 and −109/−100 bp in the −198/+88 OAT1 promoter fragment did not affect ligand‐activated ERα‐dependent OAT1 promoter activation. Additionally, studies showed that ERα can also activate gene transcription indirectly via AP1‐ or SP1‐binding sites in gene promoters (Paech et al., 1997; Safe, 2001; Kim et al., 2003). However, no DNA‐protein interaction of ERα and OAT1 promoter could be observed in EMSA, which excludes direct and indirect promoter activation of OAT1 despite predicted ER and AP1‐ or SP1‐binding sites, respectively. In an genome wide identification of high‐affinity estrogen receptor binding sites in human and mouse cells, no direct ER binding to OAT1 promoter was seen within 70,000 identified motifs (Bourdeau et al., 2004). Those data led us to the new hypothesis that ligand‐activated ERα indirectly induces OAT1 promoter by initiation of other proteins, in particular transcription factors. Subsequently, transcription factor array and proteomic analysis were performed to determine candidate transcription factors which were induced by ligand‐activated ERα in OK cells. CBF and HNRNPK were identified as candidate transcription factors in these analyses. CBF transcription factor activity was significantly increased upon 17β‐estradiol‐activated ERα incubation and also the CBF mRNA level was increased. This finding is in line with Carroll *et al.* who found CBF to be an ERα target gene (Carroll et al., 2006). In addition to increased CBF mRNA upon 17β‐estradiol incubation, an increase in endogenous OAT1 mRNA level in the used cell model (OK cells) was shown. Moreover, gene suppression experiments with siRNA targeting CBF showed a significant decrease in ligand‐activated ERα‐induced OAT1 promoter activation, suggesting a key role of CBF in OAT1 promoter regulation.

CBF, also called CCAAT enhancer‐binding protein zeta (C/EBPζ), belongs to the family of CCAAT enhancer‐binding transcription factors, harboring a highly conserved C‐terminal with a basic‐leucine zipper for dimer formation and DNA motif binding (Ramji and Foka, 2002). CCAAT is a common element of most eukaryotic promoters and CBF is highly conserved among species (Li et al., 1992; Maity and Crombrugghe, 1998). Human (NM_005760.2) and *opossum* (TRINITY_DN16007_c0_g1_i1) mRNA transcripts share 75 % similarity. Before, CBF has been shown to be involved in regulation of membrane transporters, such as multidrug resistant protein (MDR1) and a family member C/EBPα in glucose transporter (GLUT4) regulation (Kaestner et al., 1990; Goldsmith et al., 1993; Im et al., 2007). The tested human OAT1 promoter harbors a CCAAT motif −195/−190 bp before the transcriptional starting site to which CBF possibly binds to and unfolds its enhancing function.

Furthermore, HNRNPK was identified via mass spectrometry as estrogen‐induced transcription factor within the distinctly upregulated proteins. HNRNPK is a ubiquitously expressed protein with many different functions in cellular processes, e.g. chromatin remodeling, transcription, splicing, translation, and mRNA stability. The 464 aa HNRNPK protein harbors three consecutive K homology (KH) domains responsible for RNA and DNA binding, a nuclear localization signal and a nuclear shuttling domain, allowing HNRNPK to move across the cell compartments (Otoshi et al., 2015). For our study, the ability to act as transcription factor was of great interest (Michelotti et al., 1996). Genes like c‐myc, c‐Src, and the activation of the angiogenic factor VEGF have already been shown to be regulated by HNRNPK (Takimoto et al., 1993; Michelotti et al., 1996; Ritchie et al., 2003; Uribe et al., 2011).

Not only the protein expression, but also HNRNPK mRNA levels were increased in OK cells upon ligand‐activated ERα. Suppression of HNRNPK by siRNA clearly reduced the ERα‐induced OAT1 promoter activation. Nagai *et al.* already stated higher HNRNPK protein levels in ER positive breast cancer tissues compared to ER negative tumors which fits our observations of increased HNRNPK mRNA and protein level upon ligand‐activated ERα in OK cells (Nagai et al., 2004). Furthermore, Nagai *et al.* suggested, based on the correlation of HNRNPK protein level and ER positivity, that HNRNPK gene expression is regulated by ER (Nagai et al., 2004). Specific transcription factor motifs for ER, including SP1 sites, half estrogen responsive element consensus sequences, and a perfect palindromic ER binding site in the 5’ upstream of the HNRNPK gene promoter suggest a hormone dependent or independent gene activation pathway (Nagai et al., 2004). Our results clearly show that 17β‐estradiol‐induced ERα increases HNRNPK mRNA and protein level, supporting a hormone‐dependent activation of HNRNPK promoter.

Subsequently, it was investigated how HNRNPK activates human OAT1 promoter activity. Since HNRNPK DNA‐binding motifs are not yet included in binding site prediction tools, the −198/+88 OAT1 promoter fragment sequence was scanned manually for similarities to a HNRNPK‐binding site motif published by Mikula and co‐workers (Mikula et al., 2013). The scan resulted in three possible binding sites. EMSA experiments revealed that endogenous OK cell HNRNPK and exogenous HNRNPK bind the predicted binding site lying −176/−166 bp before the transcriptional starting site of OAT1 promoter. Application of a mutated oligonucleotide or usage of siRNA prior nuclear extract isolation of OK cells resulted in no or less HNRNPK‐OAT1 promoter fragment interactions, respectively. Those results clearly show HNRNPK binding to the distinct binding motif −176/−166 bp in OAT1 promoter and its positive effect on OAT1 promoter activation.

Our results show that CBF and HNRNPK are needed for OAT1 promoter activation. HNRNPK has been shown to interact with factors of the general transcription machinery like the TATA‐binding protein (TBP) and transcriptional activators like the CBF family member C/EBP (Ramji and Foka, 2002; Lu and Gao, 2016). Interestingly, both CBF and HNRNPK were also shown to be involved in transcriptional regulation of one gene, for example, the thymidine kinase (Maity and Crombrugghe, 1998; Lau et al., 2000; Mikula et al., 2013). Thus, we propose that CBF and HNRNPK interact in initiation of OAT1 promoter activity.

In summary, human OAT1 promoter was indirectly activated by ERα via the transcription factors CBF and HNRNPK. We show that CBF and HNRNPK are crucial players in OAT1 promoter activation and are inducible by ERα. Our findings show a new possible OAT1 promoter activation mechanism. Ligand‐activated ERα activates the transcription factors CBF and HNRNPK. CBF seems to activate transcription of OAT1 promoter by CCAAT‐motif binding and HNRNPK was proven to bind to a specific OAT1 promoter sequence. Our data provide novel evidence for a sex‐dependent regulation of OAT1 on transcriptional level.

## Conflict of Interests

The authors have declared that no competing interests exist.

## Supporting information




**Figure S1.** No activation of OAT1 promoter by ERα/ERβ and ERβ/ERβ. OK cells were transiently transfected with indicated human OAT1 promoter construct and (A) ERα and ERβ or (B) ERβ expression plasmid followed by incubation with 100 nM 17β‐estradiol or DMSO control for 43 h prior luciferase assay. Data are normalized to Renilla luciferase (pRL‐TK) expression, reported as fold increase over pGL3‐Enhancer and presented as mean ± S.E.M.; n = 4; *: p < 0.05; **: p < 0.01 significantly different to DMSO control using the unpaired two‐tailed t‐test.
**Figure S2.** No binding of ERα to OAT1 Promoter DNA. Biotin (B)‐labeled oligonucleotides from ‐198 to ‐53 of human OAT1 promoter were used. Boxes represent predicted ERα binding sites. ERα probe, harboring a perfect estrogen receptor binding site (ERE: 5’‐ AGGTCACTGTGACC ‐ 3’) was generated and used as positive control for formation of ERα – DNA complex. Nuclear extracts of OK cells transiently transfected with ERα and incubated with 100 nM 17β‐estradiol were mixed with indicated biotin‐labeled OAT1 promoter fragments in the presence or absence of ERα antibody. Representative EMSA showing the results of three independent experiments. p: unbound probe; s: shift; ss: supershift.
**Figure S3.** C3 promoter activation by ERα. OK cells were transiently transfected with expression plasmid for human ERα and C3 gene promoter (C3‐luc). Cells were cultured for 43 h with 100 nM 17β‐estradiol or DMSO control prior luciferase assay. Measured firefly luciferase was normalized to Renilla luciferase, data are reported as relative luciferase activity and presented as mean ± S.E.M.; n = 4; **: p < 0.01 significantly different form DMSO control using the unpaired two‐tailed t‐test.
**Figure S4.** Endogenous HNRNPK binding to OAT1 promoter is reduced in siRNA treated samples. (A) OK cells were transiently transfected with ERα expression vector and incubated with 17β‐estradiol for 43 h prior nuclear extract preparation. Biotin‐labeled B‐OAT1 (‐198/‐165) probe and a mutant version were tested in EMSA with three independent nuclear extract preparations. Nuclear extract from HNRNPK and ERα transfected cells was used. (B) OK cells were transfected with ERα expression vector and siRNA targeting HNRNPK (HNRNPK #1, HNRNPK #2) or control siRNA (siNT) and incubated with 17β‐estradiol for 43 h prior nuclear extract preparation. Biotin‐labeled B‐OAT1 (‐198/‐165) probe was tested in EMSA with two independent nuclear extract preparation sets. Nuclear extracts from cells transiently transfected with HNRNPK, ERα and siRNA transfected cells were used. p: unbound probe; s: shift; ss: super‐shift.
**Figure S5.** No binding of HNRNPK to ‐164/‐131 OAT1 promoter fragment. (A) OK cells were transiently transfected with ERα expression vector and incubated with 17β‐estradiol for 43 h prior nuclear extract preparation. Biotin‐labeled B‐OAT1 (‐164/‐131) probe were tested in EMSA with three independent nuclear extract preparations. p: unbound probe; s: shift; ss: super‐shift.
