# Supplementary material for: Estrogen receptor α (ERα) indirectly induces transcription of human renal organic anion transporter 1 (OAT1)
Source: Physiol Rep. 2019 Nov 14;7(21):e14229. doi: 10.14814/phy2.14229 (PMC6854606; doi:10.14814/phy2.14229)
Supplement: Supplementary file 1 — Figure S1. No activation of OAT1 promoter by ERα/ERβ and ERβ/ERβ. OK cells were transiently transfected with indicated human OAT1 promoter construct and (A) ERα and ERβ or (B) ERβ expression plasmid followed by incubation with 100 nM 17β‐estradiol or DMSO control for 43 h prior luciferase assay. Data are normalized to Renilla luciferase (pRL‐TK) expression, reported as fold increase over pGL3‐Enhancer and presented as mean ± S.E.M.; n = 4; *: p < 0.05; **: p < 0.01 significantly different to DMSO control using the unpaired two‐tailed t‐test. Figure S2. No binding of ERα to OAT1 Promoter DNA. Biotin (B)‐labeled oligonucleotides from ‐198 to ‐53 of human OAT1 promoter were used. Boxes represent predicted ERα binding sites. ERα probe, harboring a perfect estrogen receptor binding site (ERE: 5’‐ AGGTCACTGTGACC ‐ 3’) was generated and used as positive control for formation of ERα – DNA complex. Nuclear extracts of OK cells transiently transfected with ERα and incubated with 100 nM 17β‐estradiol were mixed with indicated biotin‐labeled OAT1 promoter fragments in the presence or absence of ERα antibody. Representative EMSA showing the results of three independent experiments. p: unbound probe; s: shift; ss: supershift. Figure S3. C3 promoter activation by ERα. OK cells were transiently transfected with expression plasmid for human ERα and C3 gene promoter (C3‐luc). Cells were cultured for 43 h with 100 nM 17β‐estradiol or DMSO control prior luciferase assay. Measured firefly luciferase was normalized to Renilla luciferase, data are reported as relative luciferase activity and presented as mean ± S.E.M.; n = 4; **: p < 0.01 significantly different form DMSO control using the unpaired two‐tailed t‐test. Figure S4. Endogenous HNRNPK binding to OAT1 promoter is reduced in siRNA treated samples. (A) OK cells were transiently transfected with ERα expression vector and incubated with 17β‐estradiol for 43 h prior nuclear extract preparation. Biotin‐labeled B‐OAT1 (‐198/‐165) probe [file PHY2-7-e14229-s001.zip › Supporting Information.docx]

**Supporting Information**

**S1 Fig. No activation of OAT1 promoter by ERα/ERβ and ERβ/ ERβ**

OK cells were transiently transfected with indicated human OAT1 promoter construct and (A) ERα and ERβ or (B) ERβ expression plasmid followed by incubation with 100 nM 17β-estradiol or DMSO control for 43 h prior luciferase assay. Data are normalized to *Renilla* luciferase (pRL-TK) expression, reported as fold increase over pGL3-Enhancer and presented as mean ± S.E.M.; n = 4; *: p < 0.05; **: p < 0.01 significantly different to DMSO control using the unpaired two-tailed *t-test.*

**S2 Fig. No binding of ERα to OAT1 Promoter DNA**

Biotin (B)-labeled oligonucleotides from -198 to -53 of human OAT1 promoter were used. Boxes represent predicted ERα binding sites. ERα probe, harboring a perfect estrogen receptor binding site (ERE: 5’- AGGTCACTGTGACC - 3’) was generated and used as positive control for formation of ERα - DNA complex. Nuclear extracts of OK cells transiently transfected with ERα and incubated with 100 nM 17β-estradiol were mixed with indicated biotin-labeled OAT1 promoter fragments in the presence or absence of ERα antibody. Representative EMSA showing the results of three independent experiments. p: unbound probe; s: shift; ss: supershift.

**S3 Fig. C3 promoter activation by ERα**

OK cells were transiently transfected with expression plasmid for human ERα and C3 gene promoter (C3-luc). Cells were cultured for 43 h with 100 nM 17β-estradiol or DMSO control prior luciferase assay. Measured *firefly* luciferase was normalized to *Renilla* luciferase, data are reported as relative luciferase activity and presented as mean ± S.E.M.; n = 4; **: p < 0.01 significantly different form DMSO control using the unpaired two-tailed *t-test*.

## S4 Fig. Endogenous HNRNPK binding to OAT1 promoter is reduced in siRNA treated samples

## (A) OK cells were transiently transfected with ERα expression vector and incubated with 17β-estradiol for 43 h prior nuclear extract preparation. Biotin-labeled B-OAT1 (-198/-165) probe and a mutant version were tested in EMSA with three independent nuclear extract preparations. Nuclear extract from HNRNPK and ERα transfected cells was used. (B) OK cells were transfected with ERα expression vector and siRNA targeting HNRNPK (HNRNPK #1, HNRNPK #2) or control siRNA (siNT) and incubated with 17β-estradiol for 43 h prior nuclear extract preparation. Biotin-labeled B-OAT1 (-198/-165) probe was tested in EMSA with two independent nuclear extract preparation sets. Nuclear extracts from cells transiently transfected with HNRNPK, ERα and siRNA transfected cells were used. p: unbound probe; s: shift; ss: super-shift.

**S5 Fig. No binding of HNRNPK to -164/-131 OAT1 promoter fragment**

## (A) OK cells were transiently transfected with ERα expression vector and incubated with 17β-estradiol for 43 h prior nuclear extract preparation. Biotin-labeled B-OAT1 (-164/-131) probe were tested in EMSA with three independent nuclear extract preparations. p: unbound probe; s: shift; ss: super-shift.
